# 6.2 MICROSTRUCTURAL IMAGING WITH ADVANCED DIFFUSION MRI METHODS – WHAT IS GAINED AND WHAT IS LOST?

**DOI:** 10.1093/schbul/sby014.019

**Published:** 2018-04-01

**Authors:** Ofer Pasternak

**Affiliations:** Harvard Medical School

## Abstract

**Background:**

Diffusion MRI is one of the important technological advances that played a crucial role in the discovery of abnormalities related to schizophrenia. While other imaging modalities focused on volumetric changes, and on functional changes (e.g., metabolic, and vascular) diffusion MRI introduced the ability to study microstructural changes. Most diffusion MRI studies focused especially on the white matter, where the diffusion tensor imaging (DTI) analysis, which by now is considered widely available and conventional, provided unique microstructural contrasts. The most important parameter has been the fractional anisotropy (FA), which was perceived as a white matter integrity measure, and sometimes as a myelin integrity measure. The simplicity of the DTI model, and its growing availability on clinical scanners resulted with a considerable body of work demonstrating reduced FA in schizophrenia, leading to new clinical hypotheses, suggesting that mental disorders may involve white matter deficiency, which in turn would lead to mis-wiring, and connectivity issues that may explain some of the unusual symptoms associated with schizophrenia. However, even though DTI measures, and specifically FA, appear to be very sensitive to subtle brain changes, these measures are not specific to any pathology. In fact, while clinical studies attempted to relate DTI measures with white matter and myelin integrity, methodological studies provided clearer evidence that such a relationship is not warranted, since DTI measures could be affected by multitude of sources. This methodological complication raised the need for more advanced microstructural imaging, which could provide superior specificity to underlying pathologies, and especially to pathologies that are related to white matter integrity and connectivity.

**Methods:**

In the recent years advanced diffusion acquisition and modeling approaches became available, leading to a significant number of studies that have applied these new tools on psychosis populations. Tools include biological model-based approaches such as free-water imaging, NODDI and permeability-diffusivity index. Other tools select model free approaches such as Kurtosis imaging, Q-space imaging, Diffusion spectrum imaging, and Generalized FA. The advanced methods provide new ways to characterize abnormalities, but at the same time, as the models become more complicated, so are the acquisitions, their length, and their sensitivity to noise. This talk will review findings from advanced diffusion MRI methods and will compare them with those obtained by the conventional DTI approach.

**Results:**

The comparison shows that while the sensitivity to identify abnormalities is not necessarily increased by the advanced methods, the fact that in some of these approaches the specificity is improved provides new insights into the nature of the underlying abnormalities. Nevertheless, even though specificity is improved, care must be taken with the interpretation of the result given the fact that diffusion MRI is an indirect measure of microstructure, limited by the assumption embedded in each model.

**Discussion:**

The emerging results present dependency on the stage of the psychosis (e.g., first episode, chronic) as well as on the age and gender of the subjects, suggesting that care must be taken in the study design, as well as in the statistical analyses performed. The findings also promote the use of multi-modal acquisitions, as well as the collection of biological, clinical and cognitive parameters. The combined information of these different domains is more likely to truly characterize the underlying abnormalities.

